# Carriage of Class 1 integrons and molecular characterization of *intI1* gene in multidrug-resistant *Salmonella* spp. isolates from broilers

**DOI:** 10.14202/vetworld.2019.609-613

**Published:** 2019-04-25

**Authors:** Renu Gupta, Sneh Lata Chauhan, Sunil Kumar, Naresh Jindal, N. K. Mahajan, V. G. Joshi

**Affiliations:** 1 Department of Veterinary Public Health and Epidemiology, College of Veterinary Sciences, Lala Lajpat Rai University of Veterinary and Animal Sciences, Hisar, Haryana, India; 2 Department of Animal Biotechnology, College of Veterinary Sciences, Lala Lajpat Rai University of Veterinary and Animal Sciences, Hisar, Haryana, India

**Keywords:** antimicrobial resistance, Class 1 integrons, phylogenetic analysis, *Salmonella*

## Abstract

**Aim:**

The present study was conducted with the following aims: (i) To screen the *Salmonella* spp. isolates recovered from suspected cases of fowl typhoid for carriage of Class 1 integrons and analyze their association with antimicrobial resistance and (ii) to carry out molecular characterization and phylogenetic analysis of Class 1 integron-integrase (*intI1*) gene.

**Materials and Methods:**

A total of 43 *Salmonella* isolates were subjected to polymerase chain reaction (PCR) assay to determine the presence of Class1 *intI1*. Differences between different serotypes in relation to their carriage of integrons and the differences between strains containing or not containing an integron and being resistant to different antimicrobials were analyzed by Fisher exact test using STATA™ (StataCorp, College Station, TX). Phylogenetic analysis was carried out using MEGA6 software.

**Results:**

Out of 43 isolates, 40 (93.02%) were found positive for Class 1 integrons. 35/40 (87.5%) *intI1*-positive isolates were multidrug resistance (MDR) (resistant to ≥4 antibiotics), which support the hypothesis of an association between the presence of Class 1 integrons and emerging MDR in *Salmonella*. There was no significant difference among isolates resistant to different antimicrobials in Class 1 integron carrying isolates and the Class 1 integron negative isolates (p<0.05). Further, there was no significant difference among different serotypes in respect of their carriage of Class 1 integrons.

**Conclusion:**

It can be concluded that the high prevalence of Class 1 integrons indicates a high potential of *Salmonella* isolates for horizontal transmission of antimicrobial genes, especially among Gram-negative organisms.

## Introduction

Integron system is one of the important dynamic mechanisms in the evolution of multidrug resistance (MDR) which helps bacteria to acquire resistance genes in novel combinations enabling them to resist several antimicrobial agents and is frequently associated with the development of MDR in Gram-negative bacteria [1]. Integrons are capable of capturing and excising gene cassettes.

There are two types of integrons, chromosomal integrons and mobile integrons (MIs). MIs are of five classes - Class 1-5 [2]. Class 1 integrons, the most common type, mostly found as part of the Tn21 or Tn402 transposon family, have been detected in bacteria in many regions and have been identified as a primary source of antimicrobial resistance (AMR) genes and suspected to serve as reservoirs capable of exchanging resistance genes in a variety of Gram-negative bacteria [3,4]. They contain a 5′ conserved segment (5′CS) and a 3′ conserved segment (3′CS) [5].

The prevalence of integrons in *Salmonella* spp. of avian origin has not been previously reported from India. The present study was conducted with the following objectives: (i) To screen the *Salmonella* spp. isolates recovered from suspected cases of fowl typhoid for carriage of Class 1 integrons and analyze their association with AMR and (ii) to carry out molecular characterization and phylogenetic analysis of integron-integrase (*intI1*) gene.

## Materials and Methods

### Ethical approval

This study did not involve any live humans or animals and therefore no ethical approval was required.

### Isolates

Sample collection, isolation, and biochemical characterization of *Salmonella* spp., serotyping, *in vitro* antimicrobial sensitivity patterns of the *Salmonella* isolates to various antimicrobials, and genomic DNA preparation has been described in our previous publication [6].

### Screening of isolates for carriage of Class 1 integrons

Out of 45 flocks investigated for fowl typhoid, 43 *Salmonella* isolates were analyzed for the presence of Class 1 integrons according to the method previously described [7], with some modifications (annealing temperature of 56°C used instead of 54°C), using primers *intI1-F “*ACGAGCGCAAGGTTTCGGT” and *intI1-R “*GAAAGGTCTGGTCATACATG.”

### Statistical analysis

A 95% confidence interval for carriage of integrons in different serotypes was calculated using STATA™. Differences between different serotypes in relation to their carriage of integrons and the differences between strains containing or not containing an integron and being resistant to different antimicrobials were analyzed by Fisher exact test, using STATA™ (StataCorp, College Station, TX).

### Sequencing of intI1 polymerase chain reaction (PCR) products

Representative PCR products were got sequenced in the university facility using an ABI 3130xL sequencer. The products were got accessioned in GenBank (accession no. MF346348.1, MF346759.1, MF346760.1, and MF346761.1).

### Phylogenetic analysis

The related sequences (23) obtained during the BLAST search were retrieved from GenBank, and multiple sequence alignment was carried along with nucleotide sequences obtained from the present study. All the 27 sequences were aligned by ClustalW in MEGA6 software. The evolutionary history was inferred using the maximum likelihood method based on the Tamura 3-parameter model. Initial tree(s) for the heuristic search was obtained automatically by applying neighbor-join and BioNJ algorithms to a matrix of pairwise distances estimated using the maximum composite likelihood approach and then selecting the topology with superior log-likelihood value. Codon positions included were 1^st^+2^nd^+3^rd^+Noncoding. All positions containing gaps and missing data were eliminated. There were a total of 290 positions in the final dataset. Evolutionary analyses were conducted in MEGA6 using 100 bootstrap replicates [8].

## Results

**Figure-1:**
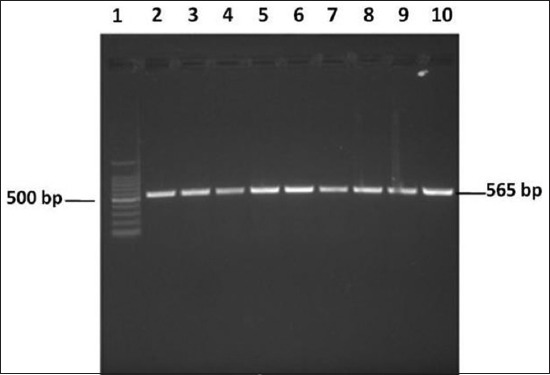
Agarose gel showing Class 1 integron integrase (intI1), amplified from *Salmonella* spp. isolates. Lane 1 – 100 bp DNA ladder; Lanes 2-10 – amplicons from *Salmonella* spp. isolates.

Of the total of 43 *Salmonella* isolates subjected to PCR assay to determine Class1 integron integrase (*intI1*), 40 (93.02%) were found positive for Class 1 integrons (Figure-1). 35/40 (87.5%) *intI1*-positive isolates were MDR (resistant to ≥4 antibiotics).

There was no significant difference among isolates resistant to different antimicrobials in Class 1 integron carrying isolates and the Class 1 integron negative isolates (p<0.05) (Table-1). Further, there was no significant difference among different serotypes in respect of their carriage of Class 1 integrons (Table-2).

**Table-1:** Number of *Salmonella* isolates with or without Class 1 integron in relation to their resistance toward different antimicrobials.

Antibiotics	Total number of resistance isolates	Class 1 Integron-positive isolates (n=40) (%)	Class 1 Integron-negative isolates (n=3) (%)
b-lactams			
Ampicillin	2	2 (5.0)	0 (-)
Carbenicillin	4	3 (7.5)	1 (33.3)
Cefotaxime	3	3 (7.5)	0 (-)
Aminoglycosides			
Gentamicin	7	7 (17.5)	0 (-)
Kanamycin	3	2 (5.0)	1 (33.3)
Streptomycin	4	4 (10.0)	0 (-)
Spectinomycin	10	10 (25.0)	0 (-)
Chloramphenicol			
Chloramphenicol	0	0 (-)	0 (-)
Tetracyclines			
Tetracycline	16	14 (35.0)	2 (66.7)
Sulfonamides			
Co-trimoxazole	13	12 (30.0)	1 (33.3)
Sulfafurazole	27	25 (62.5)	2 (66.7)
Quinolones and fluoroquinolone			
Ciprofloxacin	39	37 (92.5)	2 (66.7)
Enrofloxacin	32	30 (75.0)	2 (66.7)
Norfloxacin	32	30 (75.0)	2 (66.7)
Nalidixic acid	41	38 (95.0)	3 (100)

**Table-2:** Number of isolates of different serotypes in relation to their carriage of Class 1 integrons.

Serovar	Total number of isolates	Class 1 Integron-positive isolates	

		*n*	% (95% CI)
*Salmonella* Gallinarum	34	31	91.18 (76.3-98.0)
*Salmonella* Typhimurium	5	5	100.00 (47.8-100.0)
*Salmonella* Enteritidis	2	2	100.00 (15.8-100.0)

**Figure-2:**
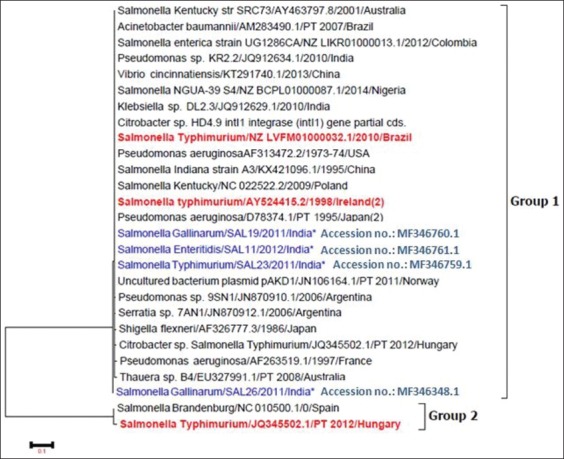
The evolutionary history of *intI1* inferred using the neighbor-joining method. The evolutionary distances were computed using the maximum composite likelihood method. The analysis involved 27 nucleotide sequences. The tree with the highest log likelihood (−879.2015) is shown. The tree is drawn to scale, with branch lengths measured in the number of substitutions per site.

Phylogenetic tree derived by analyzing and comparing the *intI1* gene sequences obtained from the current study and the published NCBI sequences is illustrated in Figure-1. Neighbor-joining phylogenetic tree indicated two distinct clads: First clad comprising all of the four isolates of the current study and almost all other isolates retrieved for analysis and the second clad comprising two isolates: *Salmonella* Brandenburg (NC 010500.1/0) from Spain and *Salmonella* Typhimurium (JQ345502.1) from Hungary (Figure-2).

## Discussion

Of the total of 43 *Salmonella* isolates subjected to PCR assay to determine Class1 integron integrase (*intI1*), 40 (93.02%) were found positive for Class 1 integrons. 35/40 (87.5%) *intI1*-positive isolates were MDR (resistant to ≥4 antibiotics). The prevalence of integrons in *Salmonella* varies from country to country and depends on the origin of the isolates as discussed below.

There are different reports of the prevalence of Class 1 integrons in *Salmonella* isolates from different parts of the world. The Overall high positive rate of 66.5% of Class 1 integrons in multidrug-resistant *Salmonella enterica* serovar Indiana (87.2%) and Enteritidis (50.8%) isolated from chickens in Eastern China has been reported [9]. Class 1 integrons were present in 35 strains (38%) of biotype *gallinarum* (90 strains) isolated from chickens in Korea [10]. The *intI1* gene was present in 31% of the *Salmonella* isolates from broiler chickens, pigs, and meat products in Thailand and Cambodia [11]. The prevalence of Class 1 integrons in *Salmonella* isolates from humans and animals in Vietnam was found to be 28% [12], whereas, in China, Class 1 integrons were detected in 10/62 (16.13%) *Salmonella* isolates from different retail foods [9]. A high prevalence of Class 1 integron in drug-resistant *Salmonella enterica* serovar Enteritidis isolates of poultry origin from Iran (17/30, 56.66%) has also been reported [13].

In our study, there was no significant difference among isolates resistant to different antimicrobials in Class 1 integron carrying isolates and the Class 1 integron negative isolates (p<0.05). Further, there was no significant difference among different serotypes in respect of their carriage of Class 1 integrons.

In contrast, a significant relation was found for the presence of Class 1 integrons and resistance to trimethoprim, sulfonamides, and tetracycline in *Escherichia coli* isolated form laying hens in Belgium [7]. Similarly, *Salmonella* isolates (recovered from retail raw chicken carcasses in China), harboring Class I integrin, presented a significantly (p<0.05) higher resistance to tetracycline, ampicillin, trimethoprim-sulfamethoxazole, amoxicillin-clavulanic acid, chloramphenicol, kanamycin, gentamicin, ceftiofur, cefoxitin, and amikacin compared with the average resistance rates [14]. The resistance of *E. coli* isolates toward sulfafurazole, trimethoprim, streptomycin, gentamicin, kanamycin, tobramycin, chloramphenicol, and amoxicillin was found to be associated with integron existence [15]. Similarly, another study has shown resistance against sulfonamides, trimethoprim, streptomycin, chloramphenicol, amoxicillin, tetracycline, and neomycin to be associated with integron existence [16]. Differences between *intI1-*positive and *intI1-*negative isolates in resistance to gentamicin, streptomycin, aminosidine, triple sulfonamides, and trimethoprim combined with sulfamethoxazole have been suggested to be significantly higher in *intI1*-positive compared with *intI1*-negative isolates [17]. This non-significant difference can be due to a lower number of isolates (3/43) without integrons obtained in this study. In our study, 35/40 (87.5%) *intI1*-positive isolates were MDR (resistant to ≥4 antibiotics) which support the hypothesis of an association between the occurrence of Class 1 integrons and emerging MDR in *Salmonella*.

The outcome of phylogenetic analyses interprets the genetic similarity of *Salmonella* isolates of the current isolates to other isolates from different parts of the world, including India (Figure-2).

## Conclusion

Based on our studies, we can conclude that the high prevalence of Class 1 integrons indicates the high potential of isolates for horizontal transmission of antimicrobial genes, especially among Gram-negative organisms. Further, elaborate study with more number of isolates would statistically improve our understanding about the role of this mechanism of resistance toward different antimicrobials, thus formulating targeted strategies for the amelioration of AMR.

## Authors’ Contributions

NKM and NJ diagnosed the disease collected samples. RG conceptualized and planned the study. RG, SLC, and SK carried out the laboratory work. VGJ carried out a phylogenetic analysis. RG analyzed the data. All authors read and approved the final manuscript.
